# MACC1 is post-transcriptionally regulated by miR-218 in colorectal cancer

**DOI:** 10.18632/oncotarget.10803

**Published:** 2016-07-23

**Authors:** Katharina Ilm, Steffen Fuchs, Giridhar Mudduluru, Ulrike Stein

**Affiliations:** ^1^ Experimental and Clinical Research Center, Charité University Medicine Berlin and Max-Delbrück-Center for Molecular Medicine in the Helmholtz Association, Berlin, Germany; ^2^ German Cancer Consortium (DKTK), Heidelberg, Germany

**Keywords:** colorectal cancer, MACC1, miR-218, epigenetic regulation, alternative polyadenylation

## Abstract

Metastasis is a multistep molecular network process, which is lethal for more than 90% of the cancer patients. Understanding the regulatory functions of metastasis-inducing molecules is in high demand for improved therapeutic cancer approaches. Thus, we studied the post-transcriptional regulation of the crucial carcinogenic and metastasis-mediating molecule metastasis associated in colon cancer 1 (MACC1). *In silico* analysis revealed MACC1 as a potential target of miR-218, a tumor suppressor miRNA. Expression of these two molecules inversely correlated in colorectal cancer (CRC) cell lines. In a cohort of CRC patient tissues (*n* = 59), miR-218 is significantly downregulated and MACC1 is upregulated compared with normal mucosa. Luciferase reporter assays with a construct of the MACC1-3′-UTR harboring either the wild type or the mutated miR-218 seed sequence confirmed the specificity of the targeting. miR-218 inhibited significantly MACC1 protein expression, and consistently, MACC1-mediated migration, invasion and colony formation in CRC cells. Anti-miR-218 enhanced the MACC1-mediated migration, invasion and colony formation. Similar findings were observed in the gastric cancer cell line MKN-45. Further, we performed methylation-specific PCR of the SLIT2 and SLIT3 promoter, where miR-218 is encoded in intronic regions. The SLIT2 and SLIT3 promoters are hypermethylated in CRC cell lines. miR-218 and SLIT2 expressions correlated positively. Methyltransferase inhibitor 5-Azacytidine induced miR-218 expression and inhibited the expression of its target MACC1. We also determined that MACC1 has alternative polyadenylation (APA) sites, which results in different lengths of 3′-UTR variants in a CRC cell line. Taken together, miR-218 is post-transcriptionally inhibiting the MACC1 expression and its metastasis-inducing abilities.

## INTRODUCTION

Metastasis associated in colon cancer 1 (MACC1) was identified through differential display RT-PCR analysis of normal colon mucosa, colorectal cancer (CRC) and respective metastasis tissue specimens in our group [1]. MACC1 induces the crucial step of carcinogenesis transition from adenoma to carcinoma in mice and human [2, 3]. In CRC and its prime metastasized organs liver and lung, MACC1 was found to be significantly upregulated when compared to mucosa and adenoma [1, 4–7]. The five year metastasis-free survival (MFS) of CRC patients with low MACC1 expression was 80%, whereas the five year MFS rate dropped down to 15% for CRC patients with high MACC1 expression. Thus, high expression of MACC1 in tumor specimens served as an independent prognostic marker for MFS [1, 8]. Since its discovery, numerous studies in a variety of different solid cancer entities such as CRC, gastric and hepatocellular carcinoma, demonstrated that MACC1 serves as a prognostic biomarker for patient tumor progression and metastasis [5, 6, 9–12].

High expression of MACC1 induced proliferation, migration and invasion of cancer cell lines and induced tumor growth and metastasis formation in mice [1, 13]. First, MACC1 was identified as transcriptional regulator of the proto-oncogene c-Met [1]. In the meantime, gain or loss of function experiments revealed additional molecules and signaling axis like Akt, E-Cadherin, c-Myc, GSK3β and caspase 3 to be regulated by MACC1 [14–18]. Therefore, MACC1 is one of the key regulators of hallmarks of cancer like cell proliferation, apoptosis, metastatic events (migration, invasion, epithelial mesenchymal translation (EMT)) and angiogenesis, as well as its role in cancer cell metabolism [1, 17–21].

microRNAs (miRs) are single-stranded RNAs of 19 to 25 nt length, which mostly bind to the 3′ untranslated regions (UTR) of classical protein coding genes and inhibit the target gene expression either by degrading the respective mRNA or by inhibiting its translation [22]. miRs are also transcriptionally regulated, for example by epigenetic modifications [23, 24]. Loss of regulation control at the transcriptional and post-transcriptional levels of MACC1 lead to its overexpression in numerous cancer entities, which causes cancer progression [11, 25–27].

miR-218 is a tumor suppressor miR, which is downregulated in different cancer entities [28]. The mature miR-218 is encoded in two precursor subtypes miR-218_1 and miR-218_2, which both are located in introns of SLIT2 and SLIT3, respectively. The expression status of miR-218 depends on the promoter activity of its host genes [29, 30]. From the literature and gene expression depository databases, like The Cancer Genome Atlas (TCGA) and Gene Expression Omnibus (GEO), we know that miR-218 is downregulated and, simultaneously, MACC1 is upregulated in different cancer entities [11, 28]. *In silico* predictions revealed that the MACC1-3′-UTR contains several predicted binding sites for the miR-218-5p specific target sequence to which it can bind via its specific seed sequence. These findings and the importance of these molecules in cancer disease motivated us to explore the role of miR-218 in the post-transcriptional regulation of MACC1. As a result of our study, we identified that MACC1 and miR-218 expression levels correlated inversely in a panel of CRC cell lines. Further, expression levels of MACC1 and miR-218 were significantly upregulated or downregulated in a cohort of CRC patient specimens, respectively. The miR-218 host genes SLIT2 and SLIT3 are hypermethylated in a panel of CRC cell lines. Overexpression of miR-218 significantly downregulated the MACC1 expression and inhibited the MACC1-induced colony formation, migration and invasion in both CRC and gastric cancer cell lines. In addition, we revealed that MACC1 possesses alternative polyadenylation (APA) sites. Taken together, these data demonstrate that miR-218 is inhibiting, at least in part, the MACC1-mediated tumor progressive events.

## RESULTS

### miR-218 expression correlated inversely with MACC1 expression in CRC cell lines

**Figure 1:**
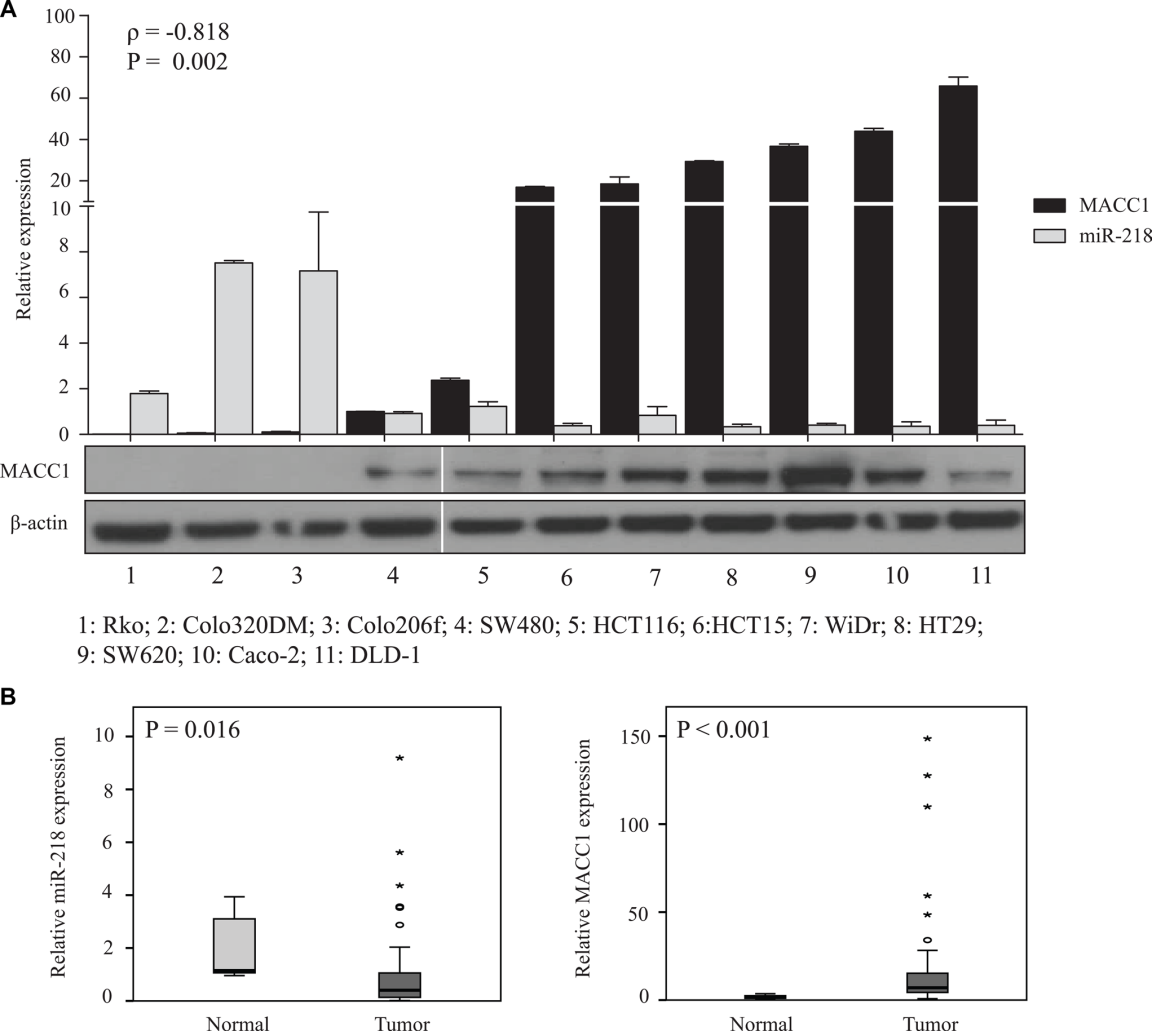
miR-218 and MACC1 expression is inversely correlated in CRC cell lines and are significantly down or upregulated in CRC tumor specimens, respectively (**A**) Relative miR-218 and MACC1 expressions in a panel of CRC cell lines were analyzed by qRT-PCR, whereas RNUB6 and RPII served as internal controls. miR-218 and MACC1 expressions are inversely correlated (*ρ* = –0.818, *P* = 0.002 ). Protein amounts of MACC1 were screened in the same panel of CRC cell lines. β-actin served as internal control. (**B**) miR-218 and MACC1 expression was measured using tumor tissue of 59 CRC patients normalized to representative normal specimens. miR-218 was significantly downregulated (*P* = 0.016) and MACC1 was significantly upregulated (*P* < 0.001) in CRC tissues. RNUB6 and RPII served as internal controls.

To investigate an *in vitro* relevance of the miR-218- and MACC1-expression in CRC cell lines, the expressions of these two molecules were measured at the transcript level. A significant inverse correlation between these two genes (*ρ* = –0.818, *P* = 0.002) was found in CRC cell lines. MACC1 mRNA and its protein amount, however, were not significantly correlated. For example, among the screened CRC cell lines, SW620 cells had the highest MACC1 protein amount and 31-fold higher mRNA expression compared to SW480 cells. Whereas, Caco-2 and DLD-1 cells had moderate endogenous protein amounts when compared with SW620 cells, but the relative MACC1 mRNA expression in Caco-2 and DLD-1 cells was higher than in SW620 cells (Figure 1A). This result shows that MACC1 mRNA expression levels not always correlate with protein expression in the analyzed CRC cell line panel.

To have further insights of the potential inverse miR-218- and MACC1-expression correlation in the clinical situation, we screened a cohort of CRC patient tumor specimens and representative normal mucosa samples. Although no significant inverse correlation was found, miR-218 was significantly downregulated and MACC1 significantly upregulated in tumor tissues compared to the normal mucosa (Figure 1B). Apart from this significant regulation, we did not find any other significant correlation of clinicopathological factors with miR-218 expression. These *in vitro* and *in vivo* expression studies of these two genes miR-218 and MACC1 prompted us to investigate the post-transcriptional regulation of MACC1 by miR-218.

### The MACC1-3′-UTR is a target for miR-218

**Figure 2:**
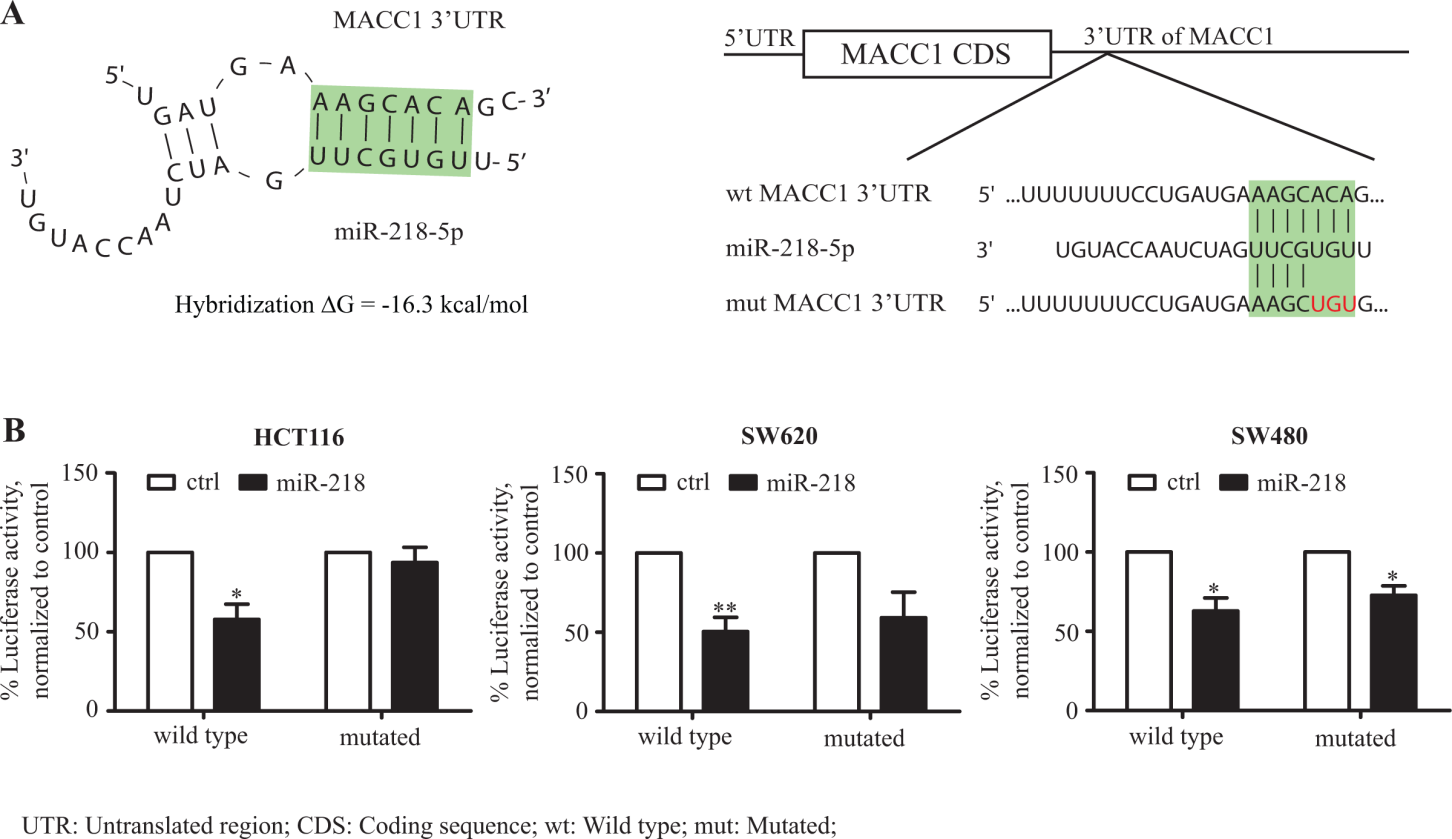
miR-218 targets the MACC1-3′-UTR (**A**) Secondary structure prediction (RNAhybrid) of the binding between the seed sequence within the MACC1-3′-UTR (position 218 to 224 nt) and miR-218 (green-highlighted sequence) revealed a hybridization energy ΔG of –16.3 kcal/mol. Representation of MACC1-3′-UTR with miR-218 binding region and schematic presentation of mutated region of miR-218 seed sequence (red-labled bases were mutated). (**B**) Luciferase reporter assays of MACC1-3′-UTR wild type and mutated construct in HCT116, SW620 and SW480. Both wild type and mutated MACC1-3′-UTR are co-tranfected either with control-miR (ctrl) or miR-218. Percent luciferase activity was calculated with the respective control (*P < 0.05; **P < 0.01).

The 6299 nt long 3′-UTR of MACC1 (#NM_182762) was screened for complementary seed sequences of known miRNAs via *in silico* prediction tools (TargetScan, RNAHybrid). A high threshold seed sequence for miR-218 at nt 218-224 was found (Figure 2A). Given the results from the expression and *in silico* analyses, we asked whether the 3′-UTR of MACC1 is a functional target of miR-218. To address this question, we cloned 6016 nt of the 3′-UTR of MACC1, harboring the miR-218 seed sequence, in pmirGLO dual luciferase vector at the 3′-position of a luciferase reporter gene (MACC1-3′-UTR). The MACC1-3′-UTR was co-transfected along with miR-218 into HCT116, SW620 and SW480 cells. The luciferase activity of miR-218-transfected MACC1-3′-UTR was significantly reduced when compared with control-miR (Figure 2B). Similarly, a co-transfection with the mutated miR-218 seed sequence construct did not show any significant reduction in luciferase activity (in HCT116, SW620) when compared with its control-miR. However, reduced luciferase activity was determined for both wild type and mutated MACC1-3′-UTR co-transfected with miR-218 in SW480, which could be due to additional miR-218 seed sequences within the 6.0 kb of MACC1-3′-UTR or miR-218-mediated secondary effects. Taken together, these data suggest that the 3′-UTR of MACC1 is a functional target for miR-218.

### miR-218 regulates the MACC1 gene expression

**Figure 3:**
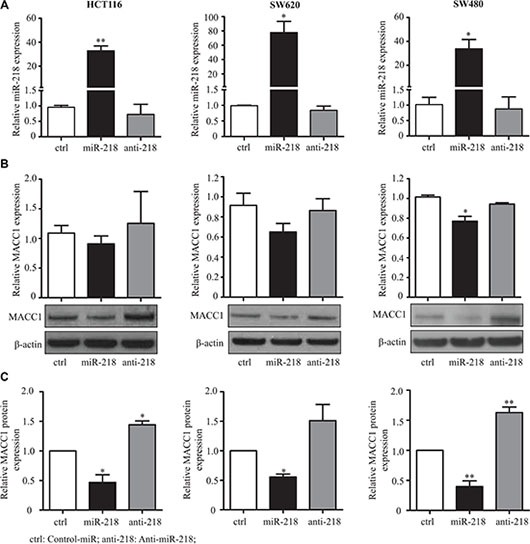
miR-218 downregulates MACC1 expression (**A**) Expression of miR-218 in the transfected conditions was quantified using qRT-PCR. RNUB6 was used as internal control. (**B**) MACC1 mRNA and protein expression after transfection of control-miR, miR-218 and anti-miR-218 was determined using qRT-pCR and Western blotting. RPII was used as internal control for MACC1 mRNA expression and β-actin for Western blotting. Either miR-218 or anti-miR-218 did not regulate the MACC1 mRNA expression significantly, but mainly regulated the MACC1 at protein level, in these three cell lines. (**C**) Densitometric analysis of MACC1 protein bands normalized to β-actin represented as a mean value of triplicates, in comparison to the respective control. (*P < 0.05; **P < 0.01).

In order to corroborate the results of the reporter assay, HCT116, SW620 and SW480 cells were transfected with control-miR, miR-218 or anti-miR-218 (complementary sequence to mature miR-218, which inhibits the function of miR-218 by complementary binding). The transfection efficiency of the miR-218 expression was measured by qRT-PCR (Figure 3A). Expression analyses showed that miR-218 reduced MACC1-transcript levels, but no changes were observed in anti-miR-218 condition, when compared with the control-miR. Western blotting confirmed the downregulation of MACC1 protein amounts in cells transfected with miR-218. On the other hand, an upregulation of the MACC1 protein amount was observed in cells transfected with anti-miR-218 in comparison to the control-miR (Figure 3B, 3C). Taken together, these results suggest that miR-218 is regulating MACC1 at the transcript level and most significantly by translation inhibition via binding to its 3′-UTR.

### miR-218 inhibits MACC1-induced colony formation, migration and invasion

**Figure 4:**
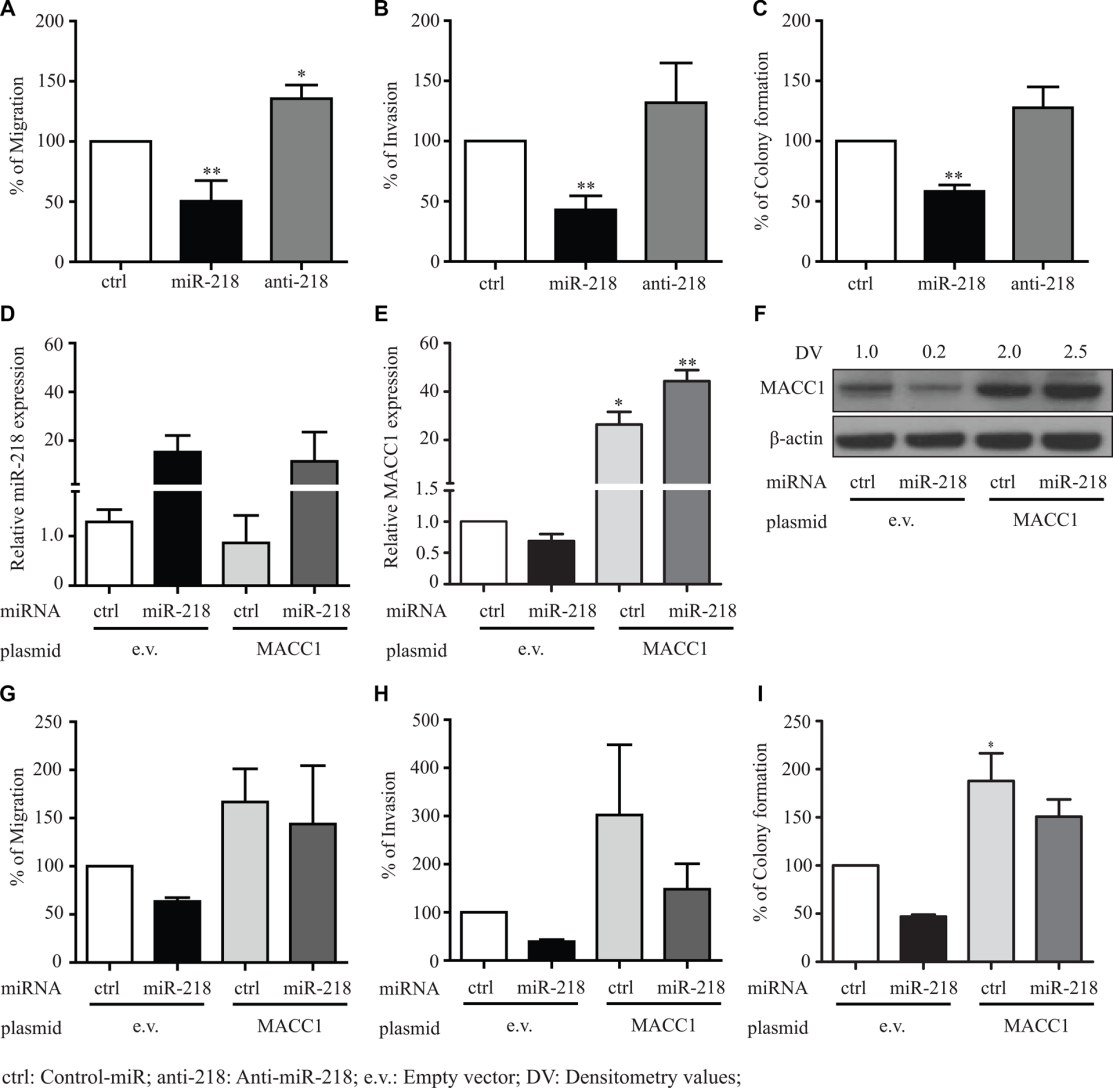
miR-218 inhibits the MACC1 mediated tumor metastasis events of CRC (**A**, **B**) SW480 cells were transfected with miR-218 or anti-miR-218. After 48 h cells were plated on top of the Boyden chambers for migration and matrigel-coated Boyden chambers for invasion. After 15 h the migrated or invaded cells were measured as described in the materials and methods. (**C**) After 48 h of respective control-miR (ctrl), miR-218 and anti-miR-218 (anti-218) transfection, cells were seeded in agar plates and counted as described in material and methods. Numbers of colonies are represented in relation with the control condition as percentage. (**D**) Relative expression of miR-218 after transfection in SW480-empty vector (e.v.) control cells and SW480-MACC1 stably expressing cells. (**E**, **F**) MACC1 mRNA and protein expression after miR-218 transfection were quantified using qRT-PCR and Western blotting. (**G**–**I**) Migration, invasion and colony formation assays of SW480-e.v and SW480-MACC1 either with control-miR or miR-218. Data are represented as the percentage of migrating or invading cells and colonies as mean ± SEM of their biological replicates, each experiment performed in three technical replicates. Densitometric analysis of MACC1 protein bands normalized to β-actin represented as a mean value of triplicates, in comparison to the respective control. (*P < 0.05, **P < 0.01, ***P < 0.001)

To further investigate the functional ability of miR-218 in mediating cancer metastatic events we have performed colony formation, migration and invasion assays by transfecting SW480 cells with either a control-miR, miR-218 or anti-miR-218 inhibitor. Colony formation assay was performed with soft agar, migration (without matrigel) and invasion (with matrigel) assays were performed with Boyden chamber transwells. Ectopic overexpression of miR-218 significantly reduced the colony formation, migrating and invading capacity of SW480 cells when compared with control-miR expressing cells (Figure 4A–4C). Ectopic overexpression of miR inhibitor anti-miR-218 significantly induced migration (Figure 4A). The cells also showed an induced invasion and colony formation when compared with the control-miR expressing cells (Figure 4B, 4C). In addition, we also investigated the effect of miR-218 on cell viability, but there was only a minor effect on proliferation detectable in SW480 cells (Supplementary Figure S1A).

Rescue experiments were performed with MACC1 stably expressing SW480 cell line with ectopic overexpression either of miR-218 or the respective control-miR along with the SW480-empty vector (e.v.) control cell line. Transfection efficiency of miR-218 expression and MACC1 expression was measured with qRT-PCR and Western blot analysis, respectively (Figure 4D–4F). In parallel to previous results with SW480 cells, in this setup the SW480-empty vector control cells showed similar results of migration, invasion and colony formation after miR-218 ectopic overexpression. As expected, the SW480-MACC1 cell line induced migration and invasion when compared to SW480-empty vector cell line (Figure 4G, 4H). miR-218 ectopic overexpression resulted in reduced migration and invasion in SW480-empty vector cells, but did not show any significant inhibition of migration and invasion in SW480-MACC1 cells, when compared with its miR-control transfected cells (Figure 4G, 4H). Similar functional effect was observed with colony formation assay (Figure 4I). Additionally, we observed increased ectopic MACC1 expression in miR-218 co-transfected cells comparative to miR-control cells. This could be due to the global effect of miR-218, which might be driving the CMV promoter or stabilizing the mRNA binding proteins. However, other possible molecular mechanisms cannot be ruled out.

Taken together, these results indicate that miR-218 significantly inhibits different steps of MACC1-mediated metastasis including migration, invasion and colony formation. miR-218 induced changes can be in part rescued by MACC1 overexpression which indicates MACC1 as novel target and mediator of the tumor suppressor miR-218 function.

### miR-218 inhibited the MACC1 expression and its mediated tumor progressive effects in gastric cancer

**Figure 5:**
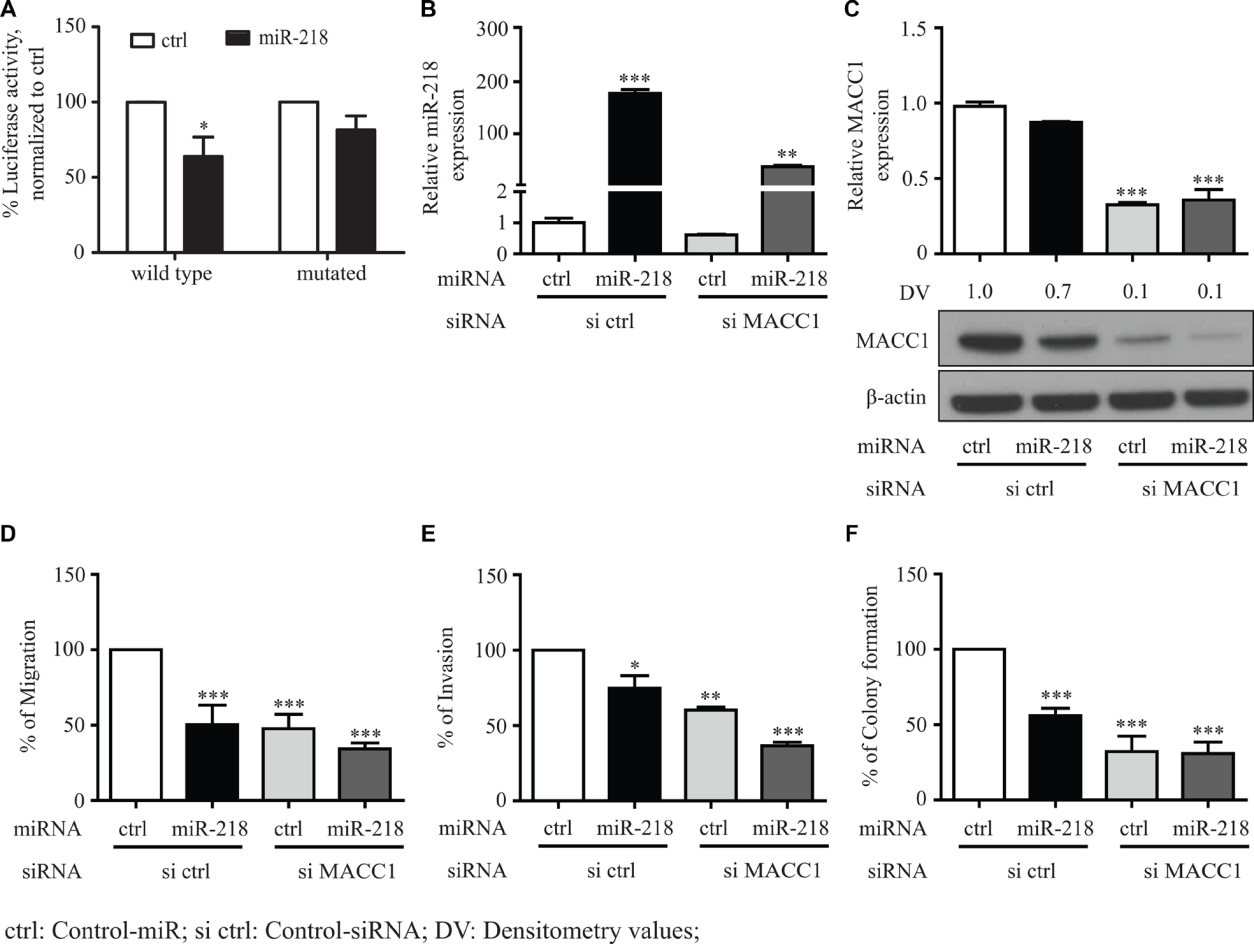
miR-218 inhibits the MACC1 mediated tumor metastasis events of MKN-45, a gastric cancer cell line (**A**) Both wild type and mutated MACC1-3′-UTR were co-transfected either with control-miR (ctrl) or miR-218 and luciferase activity were quantified. Percent luciferase activity was calculated with the respective control. (**B**) Relative expression of miR-218 after transfection in MKN-45 transfected control (ctrl), miR-218, si MACC1 and both miR-218 and si MACC1. (**C**) MACC1 mRNA and protein amounts after miR-218 and si MACC1 transfection were quantified using qRT-PCR and Western blotting. RPII was used as internal control. β-actin was used as internal control for Western blotting. (**D**–**F**) Migration, invasion and colony formation assays of MKN-45 either with control-miR (ctrl), miR-218, si MACC1 or in combination of miR-218 and si MACC1. Data are represented as the percentage of migrating or invading cells and colonies as mean ± SEM of their biological replicates, each experiment with three technical replicates. Densitometric analysis of MACC1 protein bands normalized to β-actin represented as a mean value of triplicates, in comparison to the respective control. (*P < 0.05, **P < 0.01, ***P < 0.001)

To determine in general the functional role of miR-218 for the post-transcriptional regulation of MACC1, we have used an additional cancer entity of the gastrointestinal tract, employing the gastric cancer cell line MKN-45. Consistent with our results performed with CRC cell lines, MACC1 is also a target of miR-218 in gastric cancer cells. Luciferase assay, qRT-PCR and Western blot analyses performed with MKN-45 cells revealed that MACC1 is post-transcriptionally regulated by miR-218 (Figure 5A–5C). In addition, miR-218 reduced the cell viability of MKN-45 cells (Supplementary Figure S1B). We have performed functional experiments with control-miR, miR-218, si MACC1 and both miR-218 and si MACC1. Ectopic overexpression of miR-218 or si MACC1 significantly reduced the migration, invasion and colony formation in comparison to the respective experimental controls. A combination of both miR-218 and si MACC1 further reduced migration, invasion and colony formation when compared with the individual respective control conditions. Ectopic overexpression of miR-218 and knock-down of MACC1 showed the most significant reduction when compared with experimental controls (Figure 5D–5F). This could be due to the lack of MACC1 expression as one of the important post-transcriptional targets of miR-218, indeed miR-218 could actively inhibit other targets. However, one cannot exclude other biological functions mediated due to this condition. These cumulative evidences suggest that miR-218-induced effects on migration, invasion and colony formation in different cancer entities are at least in part a result of the inhibition of the MACC1 expression.

### miR-218 is downregulated in CRC cell lines due to a promoter hypermethylation of its host genes SLIT2 and SLIT3

**Figure 6:**
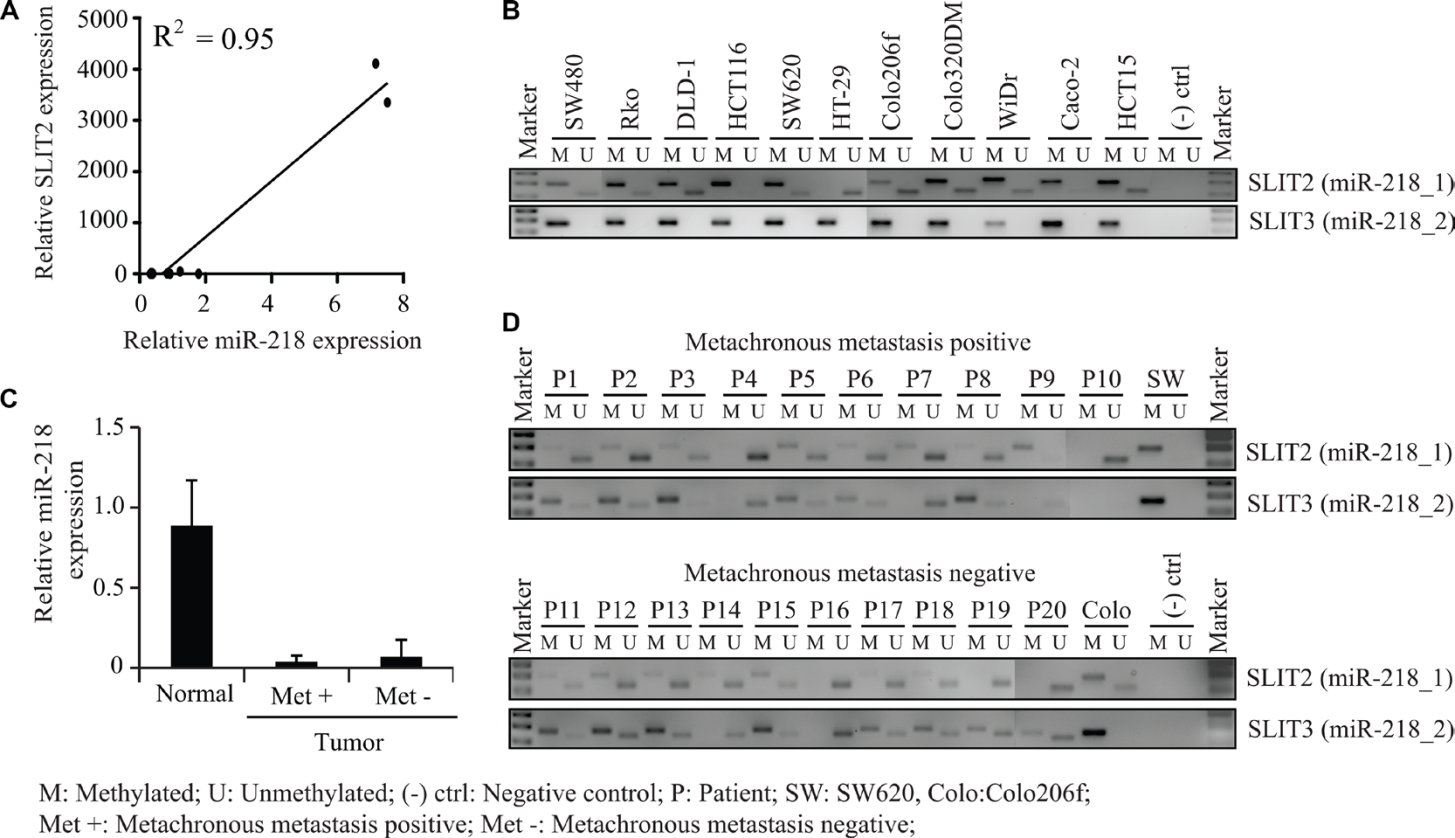
Methylation status of miR-218 host genes SLIT2 and SLIT3 promoters in CRC cells and CRC metachronous metastasis positive and negative specimens (**A**) miR-218 and SLIT2 expression were measured using qRT-PCR and positively correlated in a panel of CRC cell lines (*R*^2^ = 0.95). (**B**) Gel electrophoresis of PCR products of CRC cell lines obtained from methylation-specific PCR for miR-218_1 hosting gene SLIT2 and miR-218_2 hosting gene SLIT3. (**C**) miR-218 expression in metachronous metastasis positive and negative CRC tumor specimens in comparison to representative normal mucosa. RPII and RNUB6 served as internal controls. (**D**) Gel electrophoresis of PCR products of CRC tumor specimens obtained from methylation-specific PCR for miR-218_1 hosting gene SLIT2 and miR-218_2 hosting gene SLIT3.

As determined earlier in this study, miR-218 is downregulated in CRC cell lines and tumor specimens in comparison to normal tissue samples. Moreover, miR-218 is reported to be downregulated across different cancer entities like for example glioblastoma, nasopharyngeal and non-small cell lung cancer [28, 29, 31–33]. It is also known that miR-218 can be epigenetically silenced e.g. by promoter hypermethylation which induces esophageal carcinogenesis [34]. These reports prompted us to study the mechanisms behind the downregulation of miR-218 in CRC. With this regard, we analyzed the expression of the miR-218 and its host gene SLIT2 by qRT-PCR in our panel of CRC cell lines and found that the miR-218 expression correlated positively with the SLIT2 expression (Figure 6A). For better representation of SLIT2 and miR-218 expression, log_10_ transformed expression values were shown as Supplementary Figure S2 (*R*^2^ = 0.95; *P* < 0.001).

This made us to check methylation status of the SLIT2 and SLIT3 promoters with the published primer set by Narayan [30]. For this, DNA was isolated from a panel of CRC cell lines and subjected to bisulfite conversion and to a subsequent methylation-specific PCR. Here, primer pairs specific for the methylated or unmethylated CpG island promoter region of SLIT2 and SLIT3 were used (Figure 6B). The SLIT3 promoter was hypermethylated in all the eleven screened CRC cell lines. Moreover, the SLIT2 promoter was mostly methylated, like for example in CaCo-2 cells, which showed consequently also a relative low miR-218 expression (Figure 6B). However, there were also cell lines with a more unmethylated SLIT2 promoter like Colo206f or Colo320DM, which showed the highest miR-218 expression in the qRT-PCR comparatively (Figure 6B, Figure 1A). To sum up, the hypermethylation of the two host genes SLIT2 and SLIT3 could be the reason for the miR-218 downregulation in most of the screened cell lines.

miR-218, known to inhibit tumor metastasis formation was also significantly downregulated in CRC patient specimens [28, 29]. In this front, we wanted to investigate the clinical relevance of the SLIT2 and SLIT3 promoter methylation in metachronous metastasis positive and negative tumor specimens of CRC patients. Surprisingly, we haven't found any significant change in the methylation pattern of miR-218 host genes or in its expression between these two groups (Figure 6C, 6D). This could mean that a miR-218 downregulation might be an early event in primary tumor progression independent of metastasis formation.

### 5-Aza treatment reactivates miR-218 expression and inhibits MACC1 expression

**Figure 7:**
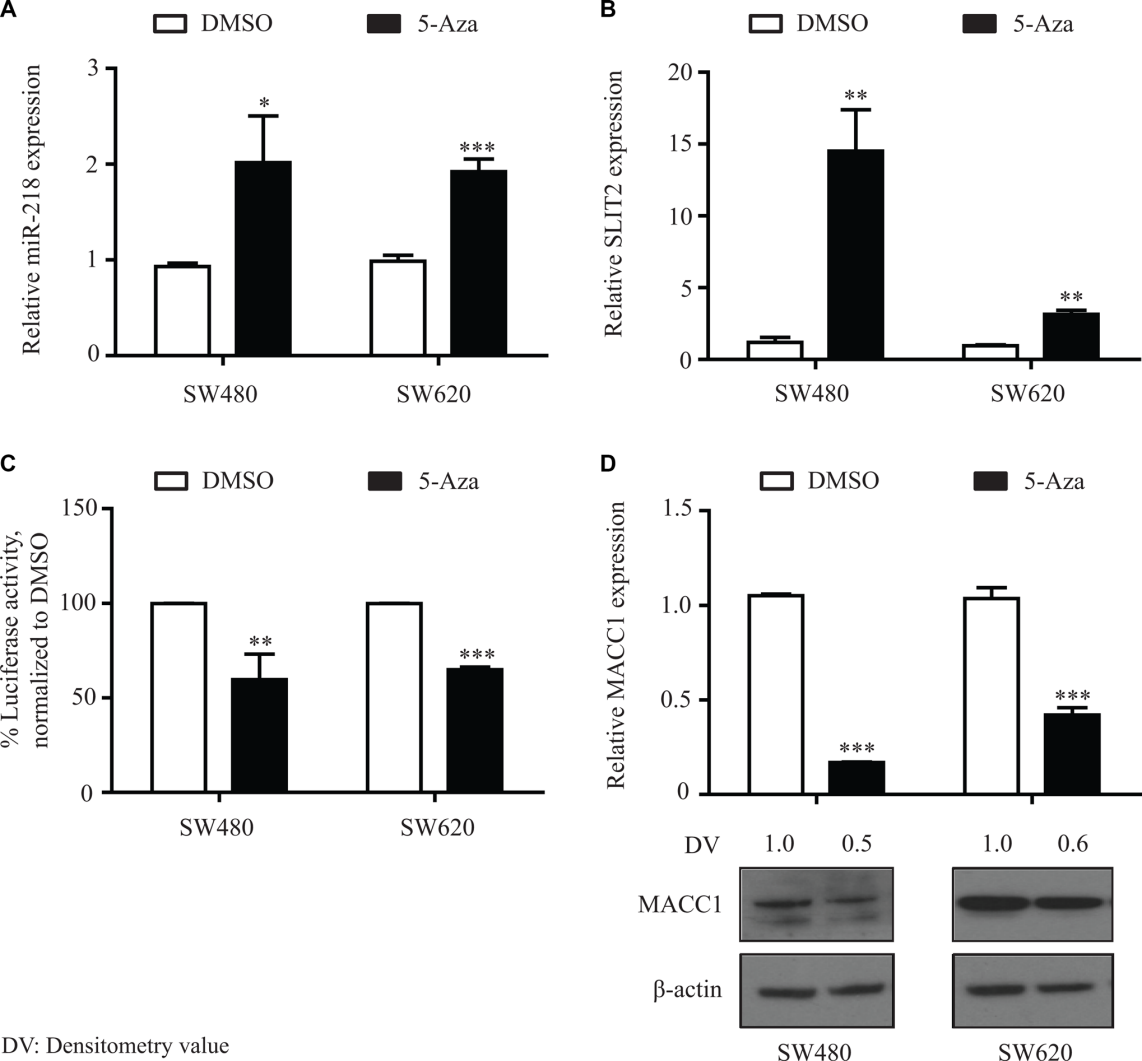
5-Aza treatment induced miR-218 expression and downregulated MACC1 gene expression SW480 and SW620 cells were treated with 5-Aza (2 μM) for 3 days and total RNA and protein were isolated. (**A**, **B**) qRT-PCR revealed induction of miR-218 and its host gene SLIT2 expression. (**C**) Luciferase activity of MACC1-3′-UTR was significantly reduced after 5-Aza (2 μM). (**D**) qRT-PCR and Western blot analysis revealed inhibition of MACC1 expression in the 5-Aza treated samples compared to DMSO treated controls. RNUB6, RPII and β-actin served as internal controls. Densitometric analysis of MACC1 protein bands normalized to β-actin represented as a mean value of triplicates, in comparison to the respective control.

In order to further differentiate the epigenetic regulation mechanism of miR-218 and MACC1, the two CRC cell lines SW480 and SW620 were treated with a selective inhibitor of DNA methyltransferases 5-aza-2′-deoxycytidine (5-Aza). Cells were treated for 72 h with 2 μM 5-Aza by changing the medium with the drug or the DMSO control every 24 h, respectively. Interesting results were observed regarding the MACC1-3′-UTR luciferase activity and the MACC1 gene expression after 5-Aza treatment. Both of the cell lines showed an induction of miR-218 and SLIT2 expression (Figure 7A, 7B). Consistently, significantly reduced luciferase activities were observed after 5-Aza treatment (Figure 7C). These observations resulted in significant downregulation of MACC1 in both cell lines at the mRNA and protein level (Figure 7D). This could be due to global effects of 5-Aza on the promoter methylation status of other miRs or transcriptional regulators that might affect the MACC1 expression. These data suggest that the epigenetic downregulation of miR-218 leads to an upregulation of MACC1 at least in part due to the loss of the miR-218 function in CRC cell lines and tumor specimens.

### MACC1 possesses alternative polyadenylation (APA) sites

**Figure 8:**
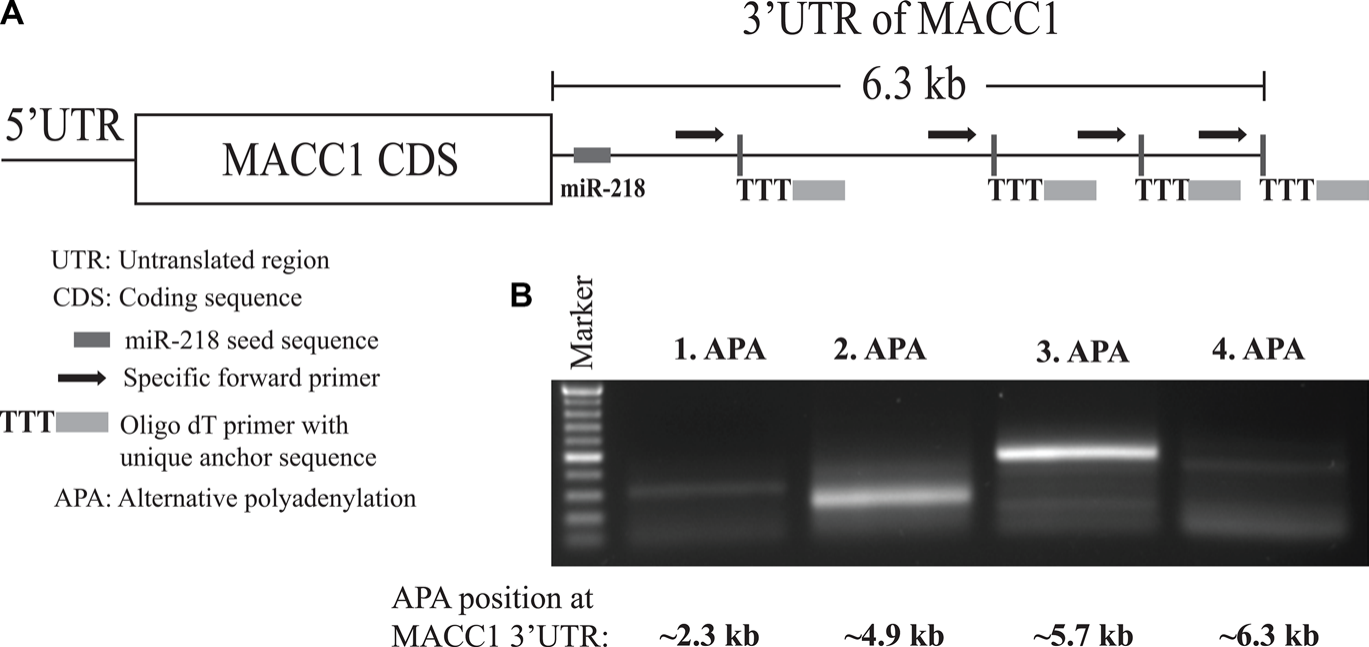
MACC1 possess alternative polyadenylation (APA) sites (**A**) Schematic presentation of MACC1-3′-UTR with miR-218 seed sequence and the different preferential APA sites. (**B**) Gel electrophoresis of PCR products obtained from 3′-RACE PCR.

Recent technical advancement of sequencing methods revealed that protein coding gene transcripts possess alternative polyadenylation (APA), and have shorter 3′-UTR both in cancer cells and in cancer specimens when compared with healthy controls [35–37]. A full length 3′-UTR of a specific gene is unavoidable for a comprehensive post-transcriptional regulation by miRNAs. Further, it is also known that some of the important cancer-related genes have shorter 3′-UTRs in cancer tissue than in normal tissues due to APA sites [35, 38]. Due to this reason and the important role of MACC1 upregulation in different cancer entities, we have investigated whether MACC1-3′-UTR contains any APA sites (Figure 8A).

We performed 3′-RACE experiment using the CRC tumor cell line SW620 and found that MACC1 possesses several APA sites in these cells (Figure 8B). SW620 cells have the highest MACC1 protein expression and transcribe the longest 3′-UTR which harbors miR-218 seed sequences. Therefore, this regulatory mechanism does not affect the post-transcriptional regulation of MACC1 by miR-218 in this cell line. However, in dealing with MACC1 APA sites a broader screening is required between different cancer cell lines and entities for better understanding of this phenomenon.

## DISCUSSION

In this study, we report that the tumor suppressor miR-218 post-transcriptionally downregulates MACC1. Expression of miR-218 is inversely correlated with the expression of MACC1 in a panel of CRC cell lines. miR-218 and MACC1 expression were significantly down- or upregulated respectively in a cohort of CRC tumor specimens. *In vitro* ectopic overexpression of miR-218 significantly inhibited the luciferase activity of MACC1-3′-UTR and MACC1 protein expression, as well as MACC1-induced migration, invasion and colony formation in CRC and gastric cancer cells. miR-218 and its host gene SLIT2 expression levels are positively correlated but there was no significant differences in miR-218 expression between metachronous metastasis positive and negative CRC tumor specimens. Secondly, miR-218 host genes SLIT2 and SLIT3 promoters are hypermethylated in the majority of CRC cell lines and tumor specimens. 5-Aza treatment significantly induced the expression of SLIT2 and miR-218 expression and inhibited the MACC1-3′-UTR luciferase activity and MACC1 expression. We also determined that MACC1 possesses alternative polyadenylation sites, which determines the availability of the 3′-UTR for the post-transcriptional regulation by miRs.

miR-218 is an intronic miR of SLIT2 and SLIT3, which are epigenetically silenced in different cancer cells and tissues [30, 34, 39]. In parallel, a downregulation of miR-218 was also reported in various cancers entities like esophageal, hepatocellular, pancreatic, gallbladder, osteosarcoma, nasopharayngeal, colorectal, non-small cell lung cancer and breast cancer, which could be due to the epigenetic silencing of its host gene in the tumor cells [28, 29, 31, 34, 40–45]. Patients diagnosed with malignant glioma, gastric or colorectal cancer showing a low miR-218 expression had a shorter disease-free survival [31, 46, 47]. On the other hand, high MACC1 expression was shown to be prognostic for shorter survival in several entities [8, 11, 48, 49]. In this present study, we also found miR-218 and MACC1 gene expression was significantly down- or upregulated, respectively, in CRC tumor specimens. In support of this inverse regulation of these two genes, luciferase reporter assay, qRT-PCR and Western blot analysis confirmed the post-transcriptional regulation of MACC1 by miR-218 in both CRC and gastric cancer cell lines.

miR-218 is a post-transcriptional regulator of the receptor Roundabout1 (Robo1) and acts via inhibiting the SLIT-Robo1-mediated tumor migration, invasion in different cancer entities [29, 47]. The epigenetic silencing of miR-218 leads to Robo1 overexpression and activation of the signaling axis after its interaction with SLIT2 [29, 50]. Along with Robo1, other molecules like receptor protein tyrosine phosphatase alpha (RPTPα), myocyte enhancer factor 2D (MEF2D), and runt-related transcription factor 2 (RUNX2) are post-transcriptional target molecules of miR-218, which are known to induce cancer metastasis like MACC1 [1, 50–52]. Rescue experiments with MACC1 overexpression or knock-down of MACC1 and miR-218 clearly revealed that MACC1 as a novel target of miR-218 is at least in part responsible for the significant reduction of migration, invasion and colony formation by miR-218 in CRC and gastric cancer cell lines [1, 15, 17]. EMT plays a major role in solid cancer tumor progression, which is triggered due to transcriptional preprograming [53]. MACC1 is known to induce c-MET as receptor tyrosine kinase involved in EMT and key transcriptional regulators like TWST1/2 [1, 54]. MACC1 is also a post-transcriptional target of another miR, namely miR-338-3p, which targets the important EMT initiating transcription factor ZEB2 [26]. Like miR-338-3p, miR-218 is also a key EMT regulator, which targets for example ZEB2 and the mesenchymal marker N-cadherin [28]. In tumor tissues, downregulation of the MACC1 post-transcriptional regulators, miR-218 and miR-338-3p, could be reasons for the MACC1 overexpression and its mediated EMT, cancer metastasis formation and drug resistance [28, 54, 55].

5-Aza is used for treatment of different types of leukemia and leads to global demethylation and activation of previously silenced gene promoters. Here, treatment of the CRC cell lines with 5-Aza showed an induction of miR-218 and SLIT2 expression. The luciferase activity of MACC1-3′-UTR, MACC1 mRNA and protein expression were reduced in 5-Aza treated samples compared to DMSO controls. These results suggest that an epigenetic inhibition of miR-218 by hypermethylation of the SLIT2 and SLIT3 promoter could be one of the reasons for MACC1 upregulation and its mediated poor survival of CRC patients and other cancer entities. Further, we haven't observed any significant difference in miR-218 expression between metachronous metastasis positive and negative specimens. This suggests that downregulation of miR-218 and MACC1 upregulation are early events during cancer development even before metastasis. In previous studies, the MACC1 expression was not tumor-stage dependent which supports our findings [1, 2, 56]. In general, our findings strongly support the existing literature that miR-218 is a tumor suppressor gene and MACC1 is an oncogene [1, 57–62].

The 3′-end of eukaryotic mRNAs possess a long stretch of untemplated adenosines termed as poly(A) tail, which plays a major role in processing of 3′-ends and stability of mRNAs [63, 64]. Recent advances in RNA sequence and transcriptome sequencing methods revealed that most of the human genes contain more than one poly(A) site [38]. The preferential poly(A) sites determines the stability, cellular localization and translational efficiency of genes, since the 3′-UTR serves as a docking site for regulatory miRNAs and RNA binding proteins [65, 66]. Lin et al. recently reported that a large fraction of mRNAs (around 30%) possess APA sites in 3′-UTRs based on the cell type [36]. Secondly, a comparison of APA sites between the cultured mammary epithelial cell line MCF10A and the breast cancer cell lines MCF7 and MDA-MB-231 revealed that cancer cells showed in general shorter 3′-UTRs [35]. This could mean that these cells harbor less miRNA binding sites, which could alter the post-transcriptional regulation of cancer-relevant genes. Similarly, MACC1 showed four APA sites in SW620 cells and among them the 5.7 kb 3′-UTR is the most prominent one. Since, it is known that MACC1 is upregulated in different cancer entities. One could speculate that in general this APA mechanism might also add regulatory complexity of this gene in tumors.

In conclusion, all these findings highlight the pivotal role of miR-218 in various aspects of MACC1 expression regulation and MACC1-mediated CRC progression. Translated into clinical practice, the determination of miR-218 and MACC1 expression in tumors could be used for better therapeutic designs. Induced MACC1 expression positively and miR-218 expression negatively mitigate the cancer stem cell functions, which are responsible for the tumor formation, growth and metastasis and resistance to chemotherapy [1, 3, 67]. Including our previous studies on MACC1 and other MACC1 and miR-218 published studies demonstrated that high MACC1 and low miR-218 expressing cancer cells are resistant to chemotherapy [20, 55, 68, 69]. These accumulated evidences strongly suggest for a miR-218 replacement therapeutic approach for MACC1 high expressing CRC patients, which might resensitize patients for chemotherapy and increases the patient survival.

## MATERIALS AND METHODS

### Cell lines, cultures and drugs

Human CRC cell lines (RKO, HCT15, Colo320DM, HCT116, SW480, SW620, HT-29, Caco-2, WiDr and DLD-1) were purchased from American Type Culture Collection (ATCC; Manassas, VA) and Colo206f from German Collection of Microorganisms and Cell culture (DSMZ Leibniz Institute; Braunschweig, Germany). The gastric cancer cell line MKN-45 was kindly provided by the Experimental Pharmacology & Oncology Berlin-Buch GmbH (EPO; Berlin, Germany). Cells were grown at 37°C with RPMI (Colo320DM, Colo206f, WiDr, SW480, HCT15) and DMEM (rest of the cells used for this study) media supplemented with 10% fetal calf serum (FCS). Original stock solutions of 5-aza-2′-deoxycytidine (5-aza-dC (#A2385), Sigma Chemical Co., St. Louis, MD) was stored at a concentration of 10 mM at –20°C and freshly dissolved in culture medium before use.

### miR, anti-miR and si-MACC1

mirVana^®^ miRNA mimic miR-218-5p (ID: MC10328), mirVana^®^ miRNA inhibitor anti-miR-218-5p (ID: MH10328), mirVana^™^ Negative Control (#4464058), Predesigned Silencer^®^ Select si MACC1 (ID:s51181) and Silencer^®^ Select siRNA negative control (#4390843) were purchased from Ambion, USA.

### Patients and samples

Fresh snap-frozen surgical specimens of tumor tissues and representative corresponding normal specimens from 59 CRC patients were collected with informed written consent (approved by Charité Ethics Committee, Charité-Universitätsmedizin, Berlin), preserved and processed as explained in our previous publications [1, 25]. The main patients' characteristics are reported in Supplementary Table S1. None of the patients received pre-operatory chemo/radiation therapy and none of the researchers conducting gene expression and statistical analyses had access to disclosed clinical-pathological data.

### Construction of 3′-UTR-luciferase plasmids and reporter assays

The 6016 bp length 3′-UTR of MACC1 was amplified using cDNA from SW480 cells and cloned into the SacI and XhoI sites of the pmirGLO dual luciferase miRNA target expression vector (#E1330, Promega, USA). With specific MACC1-3′-UTR sequencing primers, we checked for the orientation and accuracy of the 3′-UTR sequence. Specific miR-218 seed sequence were mutated using site directed mutagenesis kit (#210518, QuickChange lightning site directed mutagenesis kit, Agilent technologies, USA). Primers used for cloning, mutation and sequencing of the 3′-UTR are presented in Supplementary Tables S2, S3 and S4, respectively. For reporter assays, cells were co-transfected using Lipofectamine 2000 (Invitrogen) with 0.5 μg of dual luciferase construct along with 50 nM of control-miR or miRs/anti-miRs. Reporter assays were performed as described in the company protocol of Dual-luciferase assay-system (Promega). Briefly, after 48 h of transfection, cells were lysed using passive lysis buffer and readings were taken for luciferase and then renilla for normalization. Percentage of luciferase activity was calculated as explained before [70].

### DNA/RNA/Protein isolation and cDNA synthesis from cells and fresh snap-frozen CRC specimens

For isolation of DNA and RNA from frozen tissues, cryosections were performed and every fifth section was stained with hematoxylin. Tumor cell areas were evaluated and marked by a pathologist. Tumor cells were removed from the unstained slides and DNA was extracted by using ChargeSwitch^®^ gDNA Micro Tissue Kit (#CS11203, Thermo Fisher), according to the manufacturer's instructions. Similarly, RNA was extracted from tissues and cancer cells by using TRIzol reagent (Invitrogen). From the cancer cells, DNA was isolated using QIAamp DNA Mini Kit (#51304, Qiagen), according to the manufacturer's instructions. DNA and RNA quality and concentration were measured using the Nano drop (Thermo Scientific). 1 μg of total RNA was used to synthesize cDNA using miScript II RT Kit (Qiagen). Expression of mature miR-218 (MS00006769, Qiagen) and U6-snRNA (RNUB6) (MS00033740, Qiagen) were determined by the miScript SYBR Green PCR Kit (Qiagen), and normalized using the 2^-ΔΔCt^ method relative to RNU6B. SLIT2 (QT00007784, Qiagen), MACC1 and RPII expression were measured as described above. SLIT2 and MACC1 expression were normalized to RPII expression. Primers are provided in Supplementary Table S5.

For protein extraction, cells were washed with PBS and lysed with RIPA buffer (50 mM TrisHCl [pH 7.5], 150 mM NaCl, 1% Nonidet P-40, supplemented with complete protease inhibitor tablets; Roche Diagnostics) for 30 min on ice. Protein estimation and Western blot analysis were performed as described by Juneja et al. using specific antibodies for MACC1 (#Sigma-HPA020103) or β-actin (#SIGMA-A1978) [25].

### Cell viability, migration, invasion and colony formation assay

Cells were seeded in 6-well plates and transfected with 50 nM control, miR-218 or anti-218 (Thermo Fisher Scientific) using Lipofectamine^®^ RNAiMAX Reagent (Thermo Fisher Scientific) according to the manufacturer's instructions. Cells were seeded for cell viability, migration, invasion and colony formation assay 48 h after transfection.

For cell viability assay, 3.5 × 10^3^ cells were seeded into 96-well plates. Quantification of cell viability was achieved using MTT (final concentration 0.5 mg/ml, Sigma) colorimetric assay. The absorption values at 560 nm measured with the Infinite M200 Pro Reader (Tecan) were normalized to day 0 values and represented as fold-changes of the corresponding control. The assay was repeated at least two independent times each in triplicates.

Migration assay was performed using transwells (Costar) with 8 μm pores. 3 × 10^5^ cells were seeded per well and quantified 16 h after seeding using CellTiter-Glo^®^ Luminescent Cell Viability Assay (Promega) according to the manufacturer's instructions. For invasion assay transwells were coated with Matrigel (final concentration 10 μg/100 μl, Corning) overnight at RT and performed the same way as described for migration assay. These assays were performed three independent times, each in triplicates.

Soft agar colony formation assay was used to analyze the effects of the miRNA on anchorage-independent cell proliferation. The bottom layer contained 0.4% w/v agarose in DMEM or RPMI 1640 medium including 10% FCS and was added into 6 cm cell culture dish with grids (2 μm^2^, VWR). The top layer contained 8 × 10^3^ cells, 0.2 % w/v agarose in DMEM or RPMI 1640 medium including 10% FCS and was added onto the solidified bottom layer. Cells were seeded as single cells into the soft agar and incubated in a humidified incubator at 37°C and 5% CO_2_ for 14 days. Colonies were visualized by 10× magnification in the Zeiss AXIO microscope (Zeiss). Colonies of more than 4 cells were counted and represented as normalized to the control group. The assay was repeated three independent times.

### 5-Aza-2′-Deoxycytidine (5-aza-dC) treatment of cells, bisulfite conversion of DNA and methylation analysis

5′-aza-dC (5-Aza) treatment, bisulfite conversion and methylation analyses (PCR was performed using HotStarTaq Plus DNA Polymerase (#203605, Qiagen), were performed as described previously [70, 71]. MACC1, SLIT2 and miR-218 expression was quantified in comparison to DMSO-treated samples. CpG islands upstream of the TSS (Transcription start site) or pri-miR start site were determined with the CpG island searcher (http://www.uscnorris.com/cpgislands2/cpg.aspx), and PCR primers were designed using the Methprimer software (http://www.urogene.org/methprimer) approximately 1000 bp upstream to the SLIT2, SLIT3 and miR-218 TSS. Primer sequences are provided in Supplementary Table S6.

### 3′-Race experiment

Total RNA was isolated using the Universal RNA Purification Kit (Roboklon) according to the manufacturer's instructions including the DNase I digest step. 1 μg of RNA isolated from SW620 cells was mixed with 5 μM oligo-dT primer and 500 μM dNTPs and incubated at 65°C for 5 min. After cooling down on ice for 1 min a reaction mix for reverse transcription was prepared using 1 × RT buffer, 5 μM DTT, 40 U RiboBlock, 200 U SuperScriptIII (Thermo Scientific) and incubated at 50°C for 60 min and afterwards at 70°C for 15 min. Ampli Taq Gold (Thermo Scientific) was used for cDNA amplification according to the manufacturer's instructions. The PCR product was run on an agarose gel to visualize the different polyadenylated versions of the MACC1-3′-UTR. Primer sequences are provided in Supplementary Table S7.

### Statistical analysis

All statistical analyses were performed with IBM^®^ SPSS^®^ Statistics 21 or GraphPad Prism version 5 (La Jolla). The comparison of two different groups was done by Student's *t*-test. Comparison of three or more different treated groups was performed by one-way analysis of variance (ANOVA) and Bonferroni post hoc multiple comparison test. The non-parametric Spearmen correlation test was used for correlation analysis of MACC1 mRNA and miR-218 expression in CRC cell lines. Linear regression model was used to analyse the correlation between miR-218 and its host gene expression, SLIT2. MACC1 mRNA and miR-218 expression levels in tumor and normal specimen were examined using box and Whisker plots. Significant differences between two different groups were determined by Mann-Whitney *U*-test. All significance tests were two sided, and *P*-values less than 0.05 were defined as statistically significant.

## SUPPLEMENTARY MATERIAL FIGURES AND TABLES


