# Supplementary material for: MACC1 is post-transcriptionally regulated by miR-218 in colorectal cancer
Source: Oncotarget. 2016 Jul 23;7(33):53443–58. doi: 10.18632/oncotarget.10803 (PMC5288198; doi:10.18632/oncotarget.10803)
Supplement: Supplementary file 1 [file oncotarget-07-53443-s001.pdf]

MACC1 is post-transcriptionally regulated by miR-218 in colorectal cancer

Supplementary Materials

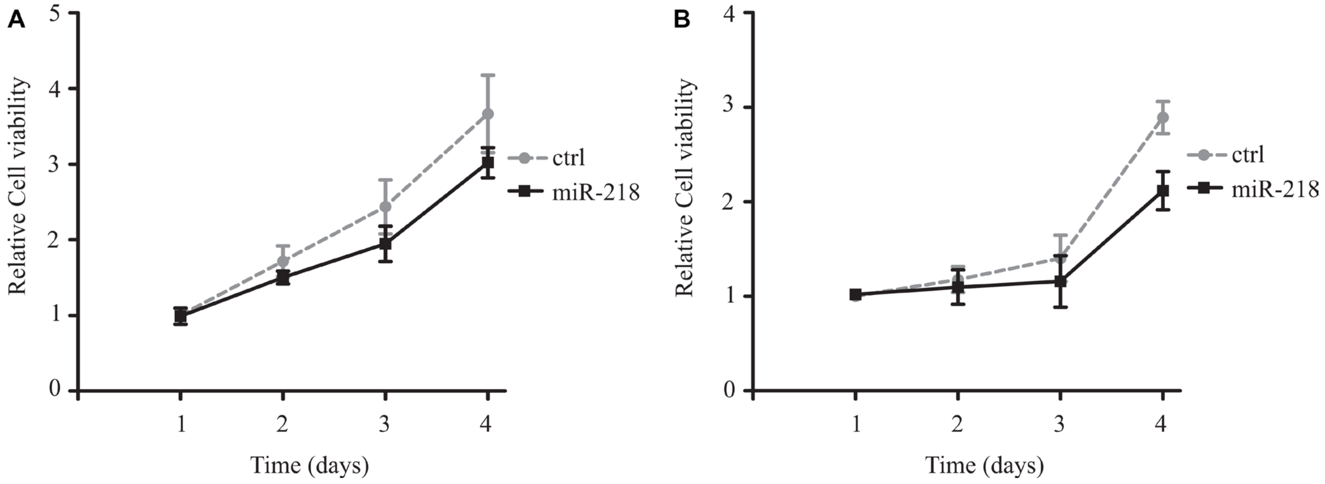

Supplementary Figure S1: Effect of miR-218 on cell viability of SW480 and MKN-45 cells. SW480 (**A**) or MKN-45 (**B**) cells were transfected either with control-miR (ctrl) or miR-218 and cell viability were determined using the colorimetric MTT assay. Absorption at 560 nm of day 0 was used for seeding normalization. Data are represented as mean ± SEM of their biological replicates, each experiment with three technical replicates.

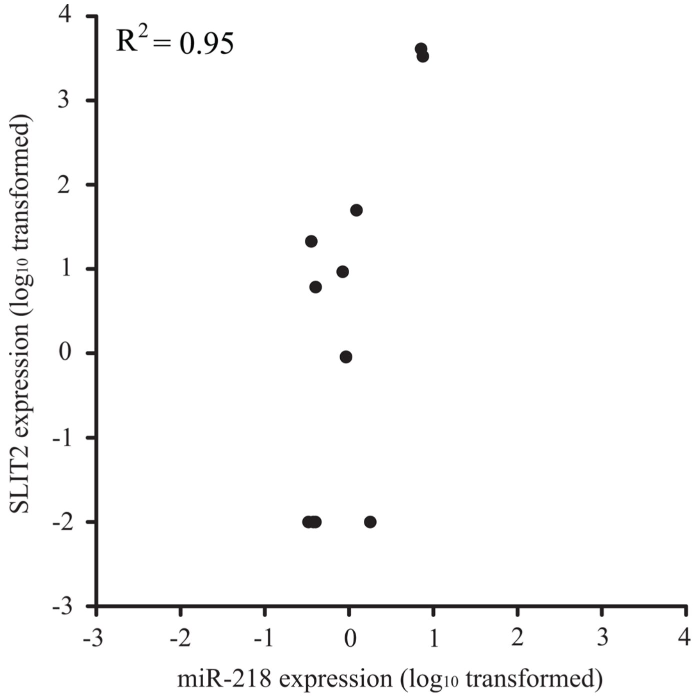

Supplementary Figure S2: SLIT2 and miR-218 expression in $\log_{10}$ scale. Expression data of all 11 cell lines included in the correlation analysis presented as $\log_{10}$ transformed scales.

Supplementary Table S1: Patients information

		Total	Percentage
**Sex**	M	36	61%
	F	23	39%
**Age median (min to max)**	63.0 (37.9 to 87.8)		
**UICC (7. Edition)**	I	17	29%
	II	22	37%
	III	20	34%
**pT**	1	6	10%
	2	16	27%
	3	27	46%
	4	10	17%
**pN**	0	39	66%
	1	14	24%
	2	6	10%
**Metachronous metastases**	no	35	59%
	yes	24	41%

Clinico-pathological features of the 59 consecutively resected CRC patients. pT = Pathologic tumor classification. pN = Pathologic lymph node status.

Supplementary Table S2: Oligos used for MACC1-3′-UTR cloning

No	Oligo Name	Sequence (5′---3′)
1	MACC1_UTR_For	AGGGAGCTCTTGATGGGAGGGAAAATGAG
2	MACC1_UTR_Rev	ATTCTCGAG TTCAGCTTGCCACCTCTTCT

Supplementary Table S3: Oligos used to mutate miR-218 seed sequences at MACC1-3′-UTR

No	Oligo Name	Sequence (5′---3′)
1	M3U_Mut_For	CAGTTTTTTTTTCCTGATGAAAGCTGTGCCTAACTGATAACCAAGATGGGT
2	M3U_Mut_Rev	ACCCATCTTGGTTATCAGTTAGGCACAGCTTTCATCAGGAAAAAAAAACTG

Supplementary Table S4: Oligos used for sequencing the MACC1-3′-UTR

No	Oligo Name	Sequence (5′---3′)
1	MACC1_Seq_1	GGAAACAGTTGCCGTTTCAT
2	MACC1_Seq_2	AGTTGCAATGGAGAGGCTGT
3	MACC1_Seq_3	CTTGCAGTGAGCCGAGTTC
4	MACC1_Seq_4	CCTTGTCCGGGTAATTTTTG
5	MACC1_Seq_5	GCATTGGGCTGAGAAACACT
6	MACC1_Seq_6	TGGTCCACAGCACAAAATAAA
7	MACC1_Seq_7	AAGGCATAGCACATTTAGCACA

Supplementary Table S5: Oligos used for MACC1 and RPII screening by qRT-PCR

No	Gene Name	Sequence (5′---3′)
1	MACC1_For	TTCTTTTGATTCCTCCGGTGA
2	MACC1_Rev	ACTCTGATGGGCATGTGCTG
3	RPII _For	GCACCACGTCCAATGACAT
4	RPII _Rev	GTGCGGCTGCTTCCATAA

Supplementary Table S6: Oligos used for methylation-specific PCR of MACC1, SLIT2 and SLIT3

No	Gene Name	Sequence (5′---3′)
1	SLIT2_M_F	GGGAGGCGGGATTGTTTAG
2	SLIT2_M_R	CATAACGCGCGAAAATACAC
3	SLIT2_U_F	GTGGGAGGTGGGATTGTTTA
4	SLIT2_U_R	ACCTCTCCCTCACCCTCAAC
5	SLIT3_M_F	GGTTTCGTCGATGGAGTTGT
6	SLIT3_M_R	AAACGCGTAAAACCCGAAA
7	SLIT3_U_R	TGTGGGTTAGTGGGGTTAGG
8	SLIT3_U_R	CACAAACAAAACAAAACACTCCA

Supplementary Table S7: Oligos used for generating cDNA of different poly-A tails in MACC1 mRNA and amplification of alternative polyadenylation (APA) sites

No	Oligo Name	Sequence (5′---3′)
1	Oligo(dT) Primer	GCGCATCGATCGGCCGCGTTTTTTTTTTTTTTTTV
2	Anchor Primer_R	GCGCATCGATCGGCCGCG
3	First_APA_F	TGT GGG TAA GCC AAA GAA GG
4	Second_APA_F	CAGCATGAAAGGCTTCCTCT
5	Third_APA_F	CCAAACATGCTCCAGTCTCA
6	Fourth_APA_F	TGCCCTGCTTTCACTGTAGA
